# Sodium Alginate/Chitosan-Coated Liposomes for Oral Delivery of Hydroxy-α-Sanshool: In Vitro and In Vivo Evaluation

**DOI:** 10.3390/pharmaceutics15072010

**Published:** 2023-07-24

**Authors:** Fengming Tan, Huan Li, Kai Zhang, Lulu Xu, Dahan Zhang, Yang Han, Jing Han

**Affiliations:** 1 Department of Pharmaceutical Engineering, Shenyang Pharmaceutical University, No. 103, Wenhua Road, Shenyang 110016, China; 2 School of Chinese Materia Medica, Shenyang Pharmaceutical University, No. 103, Wenhua Road, Shenyang 110016, China; 3 Faculty of Functional Food and Wine, Shenyang Pharmaceutical University, No. 103, Wenhua Road, Shenyang 110016, China

**Keywords:** hydroxy-α-sanshool, modified liposome, pH-response

## Abstract

Background: Hydroxy-α-Sanshool (HAS) possesses various pharmacological properties, such as analgesia and regulating gastrointestinal function. However, the low oral bioavailability of HAS has limited its oral delivery in clinical application. Methods and Results: To enhance its oral bioavailability, a nanocomposite delivery system based on chitosan (CH, as the polycation) and sodium alginate (SA, as the polyanion) was prepared using a layer-by-layer coating technique. The morphology, thermal behavior and Fourier transform infrared spectrum (FTIR) showed that the obtained sodium alginate/chitosan-coated HAS-loaded liposomes (SA/CH-HAS-LIP) with core-shell structures have been successfully covered with polymers. When compared with HAS-loaded liposomes (HAS-LIP), SA/CH-HAS-LIP displayed obvious pH sensitivity and a sustained-release behavior in in vitro studies, which fitted well to Weibull model. In vivo, the half-life of HAS from SA/CH-HAS-LIP remarkably extended after oral administration compared to the free drug. Additionally, it allowed a 4.6-fold and 4.2-fold increase in oral bioavailability, respectively, compared with free HAS and HAS-LIP. Conclusions: SA/CH-HAS-LIP could be a promising release vehicle for the oral delivery of HAS to increase its oral bioavailability.

## 1. Introduction

Hydroxy-α-Sanshool (HAS, C_16_H_25_NO_2_, M = 247.37) is lipophilic hydroxyalkamide in Zanthoxylum piperitum, which has been used as food additive and traditional medicine [1]. Because of its various pharmacological effects, HAS has been generally studied, including antioxidant, anti-inflammatory and anti-diabetic effects [2,3,4,5]. Recently, the pharmacological properties of HAS on analgesic, promoting gastrointestinal motility as well as ameliorating learning and memory, have caused many concerns [6,7,8,9]. Due to its long unsaturated chain, HAS has low solubility in water and poor stability in low pH conditions, resulting in decreased absorption in the gastrointestinal tract and the low oral bioavailability of HAS [10]. After oral administration, HAS is rapidly absorbed in the gastrointestinal tract and distributed throughout the body as glucuronide conjugates, along with low blood concentrations of the drug, which might be not enough to exert its pharmacological activities [11]. Additionally, the half-life of HAS is about 1.6–1.7 h, which may result in short action time during treatment [12]. These disadvantages have limited the clinical application of HAS for the treatment of various gastrointestinal disorders. Therefore, it is necessary to design a proper drug delivery system to gain improvement in the solubility and oral bioavailability of HAS.

Oral administration has been one of the most preferred routes for drug delivery because of its safety and convenience, which could allow more flexibility in dosing frequency and improve patient compliance [13]. Nowadays, different delivery carriers for HAS have been reported, such as nanostructured lipid carriers and liposomes, which could enhance the solubility, stability or bioavailability of HAS [14,15]. Although these delivery carriers for HAS possesses various advantages, the oral delivery of HAS remains a major challenge and often causes a decrease in oral bioavailability because of the rapid degradation in the complex gastrointestinal environment [10]. In recent years, in order to overcome these obstacles, many attempts have been made, mainly including nanocomposite delivery systems (such as the surface functionalization of liposomes), which can provide different physicochemical and functional properties for oral delivery [16]. For example, chitosan-coated liposomes with a high mucoadhesive property showed an improvement in the permeability and oral bioavailability of drugs [17]. Modified liposomes with sodium alginate and chitosan derivatives not only improved the stability of drugs but also presented sustained release behavior, which has the potential for oral delivery [18].

In this paper, to enhance the solubility and bioavailability of HAS, sodium alginate/chitosan-coated Hydroxy-α-Sanshool-loaded liposomes (SA/CH-HAS-LIP) were firstly prepared for oral application. The physicochemical characteristics and release behavior of SA/CH-HAS-LIP were analyzed to investigate the influence of chitosan (CH) and sodium alginate (SA) on liposome formulation. In addition, in vivo pharmacokinetic properties of SA/CH-HAS-LIP were studied compared to free HAS and HAS-loaded liposomes (HAS-LIP).

## 2. Materials and Methods

### 2.1. Chemicals and Animals

Hydroxy-α-sanshool standard (HAS, 98% purity) and sodium alginate (SA, low viscosity, 12 kDa) were acquired by Aladdin Corporation (Shanghai, China). Hydrogenated soybean phosphotidylcholine (HSPC) and chitosan (with a degree of N-deacetylation ≥95% and viscosity 100–200 mpa·s) were acquired from Macklin (Shanghai, China). Cholesterol was acquired from Tianjin Guangfu Fine Chemical Research Institute (Tianjin, China). All other reagents and chemicals were of analytical purity.

Healthy male Sprague Dawley rats (SD, 250–300 g) were acquired from the Experiment Animal Center of Shenyang Pharmaceutical University (Shenyang, China) and housed in an air-conditioned room at 25 ± 2 °C with free access to food and water. The procedure for the experimental animals in this study was followed in concordance with the protocol on animal experiments issued by the Shenyang Pharmaceutical University Ethics Committee (protocol code SYPU-IACUC-2018-0009).

### 2.2. Preparation of HAS-LIP

The thin-film hydration ultrasonic technique was used to prepare HAS-LIP [19]. Briefly, HAS, cholesterol and HSPC in a mass ratio of 1:1.5:21 were dissolved in the mixture of chloroform and ethanol (2:1, *v*/*v*). This solution was evaporated to form a dried thin film at 35 °C under vacuum. Then, the lipid film was hydrated by phosphate-buffered saline (PBS, pH 7.4) at 1400 rpm and subsequently sonicated (340 W, 2 s on, 8 off) using a Scents-IID probe sonicator (Scientz, Ningbo, China) for 15 min. Finally, the dispersion was dried and stored at 4 °C for further characterization.

### 2.3. Layer-by-Layer Coating of SA/CH-HAS-LIP

**Figure 1:**
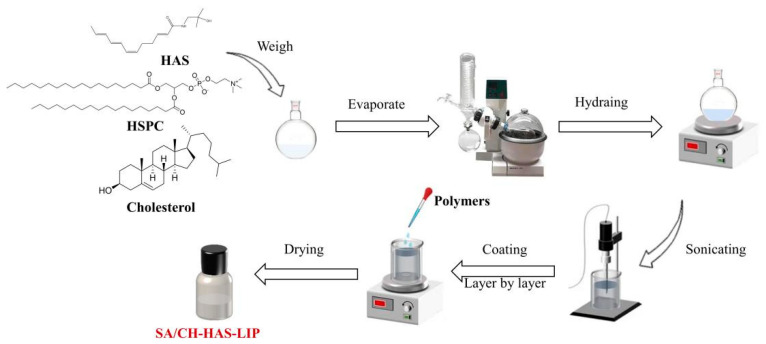
Schematic illustration of the preparation of SA/CH-HAS-LIP.

SA/CH-HAS-LIP was prepared by the layer-by-layer coating technique using CH as the polycation and SA as the polyanion (Figure 1). Briefly, the HAS-LIP suspension was dispersed in a 0.6% CH solution (1:1, *v*/*v*, dissolved in ultrapure water and adjusted to pH 5.5) with continuous stirring for 1 h, followed by 7 min of sonication (210 W, 2 s on, 8 off). To obtain the second coating layer, chitosan-coated HAS-loaded liposome (CH-HAS-LIP) dispersion was added dropwise to 0.4% SA solution (1:1, *v*/*v*, dissolved in ultrapure water and adjusted to pH 5.5) and stirred for 1 h. After that, the mixture was adjusted to a pH of 5.5 and centrifuged at 3000× *g* using a H1850 centrifuge (Cence, Changsha, China) for 10 min to remove aggregation. The obtained CH-HAS-LIP and SA/CH-HAS-LIP dispersions were dried and stored at 4 °C for further characterization, respectively. The concentrations of CH and SA were optimized by the parameters of particle size, polydispersion index (PDI), zeta potential and encapsulation efficiency (EE%), respectively. The optimization of CH-HAS-LIP and SA/CH-HAS-LIP formulations is described in the Supplementary Materials.

### 2.4. Determination of Particle Size, Polydispersity Index and Zeta Potential

The average particle size and polydispersity index (PDI) of nano-particles were determined by the dynamic light scattering (DLS) method, and the zeta potential (ζ) was measured by the electrophoretic light scattering method. Samples were diluted 100 times in PBS and taken into sample cells (DTS0012 cuvettes or DTS1070 folded capillary cells, Malvern, UK). Samples were determined at 25 °C using a Zetasizer Nano ZS 90 Nanoparticle size Analyzer (Malvern Instruments, Malvern, UK). The intensity was detected at an angle of 90°. Three parallel experiments were performed with each sample.

### 2.5. Determination of Encapsulation Efficiency and Drug Loading

The encapsulation efficiency (EE%) of liposomes was carried out by the ultrafiltration method [20]. Briefly, 500 μL of liposomes suspension was taken into a 0.5 mL centrifugal filter tube (100 kDa, Millipore, Billerica, MA, USA) and centrifuged at 5000× *g* for 15 min. The supernatant was collected, diluted to 5 mL with methanol and detected by HPLC (Hitachi, Shiga, Japan) at 254 nm. The mobile phase containing methanol and water (70:30 *v*/*v*) was pumped through a Promosil C_18_ column (4.6 × 250 mm, 5 μm, Agela Technologies, Tianjin, China) at a constant flow rate of 0.8 mL/min. The column temperature and injection volume were 40 °C and 10 μL, respectively. The regression equation was y = 2415.09 x + 1989.87 in a range of 10.0–200.0 μg/mL with a good linear relationship (R^2^ = 0.9997). The *EE*% and drug loading (*DL*%) were calculated by the following equations:

(1) $$EE\%=\frac{W_{T}-W_{F}}{W_{T}}\times 100\%,$$

(2) $$DL\%=\frac{W_{T}-W_{F}}{W},$$

where *W_T_* is the total mass of HAS in the liposome suspension (mg); *W_F_* is the mass of free HAS in the filtrate (mg); *W* is the mass of the freeze-dried liposome formulation (mg). All experiments were measured in triplicate.

### 2.6. Transmission Electron Microscopy

Transmission electron microscopy (TEM) was preformed to assess the shape and surface morphology of the formulations [21]. Briefly, samples were fixed on the microscopic carbon-coated grids and negatively stained with 2% phosphotungstic acid. Then, the images were observed using a JEM1200EX TEM (JEOL, Tokyo, Japan) at 200 kV.

### 2.7. Differential Scanning Calorimetry

To investigate the thermal behavior of samples, differential scanning calorimetry (DSC) was carried out in a DSC3 differential scanning calorimeter (Mettler Toledo, Stockholm, Sweden). Briefly, samples (5 mg) were fixed to aluminum pans and then heated from 25 to 300 °C at a constant rate of 10 °C/min under a nitrogen atmosphere (20 mL/min).

### 2.8. Fourier Transform Infrared Spectrum Analysis

Fourier-transform infrared (FTIR) spectrum analysis was adopted for the study of the possible interaction between liposomes and the coating layer. FTIR spectroscopy was performed with the KBr pellet technique. Briefly, a proper number of freeze-dried samples were fully mixed with KBr in a mass ratio of 1:200, pressed into discs and then placed in the sample chamber for testing. The scan rate was 0.5 cm/s. FTIR spectra were determined over the wavelength range of 400 to 4000 cm^−1^ using a FTIR-650 FTIR spectrometer (Tianjin Guangdong Sci. & Tech. Development Co., Ltd., Tianjin, China).

### 2.9. In Vitro Drug Release Investigation

The in vitro release study of liposomes was investigated by simulated gastric fluid (SGF) and a simulated intestinal fluid (SIF) model [22]. Briefly, samples were mixed with SGF or SIF at a ratio of 1:4 (*w*:*v*). The mixture was added into a dialysis bag (Mw = 10 kDa, Thermo Fisher Scientific, Waltham, MA, USA). Then, the end-sealed dialysis bags were incubated in 50 mL of digestive juice (SGF/SIF) at 95 rpm and 37 °C, using an SHZ-B water-bathing constant temperature shaker (Shanghai Boxun Biomedical Instrument Corporation, Shanghai, China), respectively. A total of 1 mL of the sample was collected from the receptor chamber at predetermined time intervals (0, 0.5, 1, 2, 4, 6, 8, 12, 24 h), diluted with methanol and analyzed by HPLC. Meanwhile, an equal volume of fresh release media was added immediately. All experiments were conducted in triplicate.

#### Release Mechanism

To understand the mode of HAS release to form a liposome, in vitro release data were fitted by various kinetic models (such as zero-order, first-order, Higuchi, Ritger-peppas and the Weibull kinetic model) and analyzed by a model-dependent approach using Origin software version 10.0 [23].

### 2.10. In Vivo Pharmacokinetic Assessment

#### 2.10.1. Experimental Protocol and Sampling

To evaluate the pharmacokinetic behavior of HAS in different formulations of liposomes, SD rats were randomly divided into four groups (*n* = 6), fasted for 12 h and received the oral version of HAS (10 mg/kg as a control), HAS-LIP (10 mg/kg of HAS) and SA/CH-HAS-LIP (10 mg/kg of HAS). After oral administration, blood samples (0.4 mL) were collected from the retro-orbital sinus at the scheduled time points and centrifuged (4000× *g*, 15 min) to obtain plasma. The plasma samples were stored at −80 °C until analysis.

#### 2.10.2. Blood Processing

Liquid–liquid extraction was used to separate HAS from plasma samples. Briefly, 100 μL of the plasma sample and 10 μL of methanol were taken into centrifuge tubes (Bkmam, Changde, China) and thoroughly mixed by vortexing for 30 s using an MX-S vortex shaker (Scilogex, Rocky Hill, CT, USA). Then, 2 ML of ethyl acetate was added. After vortex mixing (5 min) and centrifugation (16,500× *g* rpm, 5 min), the supernatant was collected and nitrogen-dried at 35 °C. The residue was dissolved with 100 μL of methanol, vortexed (2 min) and centrifuged (16,500× *g*, 5 min). Finally, 20 μL of clean supernatant was transferred to a UPLC-MS/MS system for analysis.

#### 2.10.3. Chromatographic and Mass Spectrometric Conditions

The plasma contents of the drug were determined using an LCMS-8050 ultra-performance liquid chromatography-tandem mass spectrometer (UPLC-MS/MS, Shimadzu, Kyoto, Japan) [24]. An ACQUITY UPLC BEH C_18_ column (2.1 × 100 mm, 1.7 μm Waters, Taunton, MA, USA) was used. The mobile phase consisted of acetonitrile (solution A) and 0.1% formic acid in water (*v*/*v*), and the gradient elution program started at 35% A (holding for 3 min), increased linearly to 65% A (holding for 1 min) and then returned to the initial 35% A (holding for 1 min). Other conditions were as follows: column temperature (35 °C), flow rate (0.3 ML/min) and injection volume (5 μL).

The mass spectrometer was conducted In the positive ionization mode using an ESI source. Detection and quantification were operated in a multiple-reaction monitoring (MRM) mode. The detection of tandem mass spectrometry was carried out by monitoring the fragmentation of 286.2→179.3 (*m*/*z*). The optimized MS conditions were as follows: capillary voltage (4 KV), gas temperature (300 °C) and gas flow (10 L/min).

### 2.11. Data Analysis

All data were expressed as mean ± SD, and the plasma drug concentration-time curve was plotted using Origin 10.0 (Microcal Software, Inc., Tempe, AZ, USA). The statistical differences between different groups were evaluated by student’s *t*-tests using SPSS 15.0 (SPSS Inc., Armonk, NY, USA), and *p* < 0.05 was considered statistically significant. The pharmacokinetic parameters of each group were performed using Drug and Statistic (DAS) 2.1.1 (provided by Anhui Provincial Center for Drug Clinical Evaluation, Changsha, China), including the half-life in the distribution phase (t_1/2α_), the elimination half-life (t_1/2β_), the attain peak concentration time (t_max_), the peak concentration (C_max_), the area under the plasma drug concentration-time curve (AUC) and the mean residence time.

## 3. Result and Discussion

### 3.1. Determination of Particle Size, PDI and Zeta Potential

Controlling the particle size of liposomes in a suitable range could be the key to prepare oral liposomes for better release amount and bio-availability. The particle sizes of HAS-LIP, CH-HAS-LIP and SA/CH-HAS-LIP were 107.81 ± 2.11, 212.32 ± 7.73 and 533.71 ± 12.39 nm, respectively (Table 1). There was a progressive increase in particle size after the liposome was coated by the CH and SA modification layer by layer. This could preliminarily demonstrate that CH and SA were wrapped on the surface of the liposome. Compared to the PDI of HAS-LIP (0.15), the PDI of CH-HAS-LIP and SA/CH-HAS-LIP increased by 0.11 and 0.21, respectively, which might be attributed to the bridging flocculation of charged polyelectrolytes [25].

Zeta potential presents the potential charge of liposomes on the near surface. In Figure S1b,d, the zeta potentials of HAS-LIP, CH-HAS-LIP and SA/CH-HAS-LIP were −58.20 ± 3.4, 56.10 ± 4.29 and −49.40 ± 6.72 mV, respectively. The negative charge of uncoated liposomes would be due to the phosphate groups in HSPC. After covering them with the CH modification, the zeta potential value changed to positive, which might be attributed to the fact that CH with a positive charge was absorbed on the surface of particles by electrostatic interaction. Then, the charge on CH-HAS-LIP was generally neutralized and changed to negative by the addition of SA as a final coating layer with a great deal of negative charge at the pH of 5.5. The change of the zeta potential could further confirm that the liposomes were successfully coated with CH and SA by layer-by-layer coating technology. This is consistent with a previous study [26]. Additionally, the absolute value of the charge of particles decreased when liposomes were subjected to layer-by-layer coating with electrically charged polymer, which may be caused by the shielding effect of charged polymers.

### 3.2. Determination of Encapsulation Efficiency and Drug Loading

EE% and DL% are crucial parameters that present the quality of liposome. As shown in Table 1, the EE% and DL% of all liposomes were higher than 92% and 2.9%, respectively. This is most likely attributed to the interaction between the drug and lipid bilayer [27]. Compared with uncoated liposomes, the slight decrease of EE% and DL% in modified liposomes were within the experimental errors. The discrepancy might be due to the consequence of CH (with a positive charge) and HAS (with a positive charge) competing for phospholipids (with a negative charge) [28].

### 3.3. Transmission Electron Microscopy

**Figure 2:**
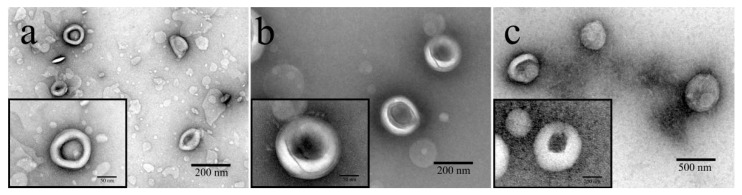
TEM images of HAS-LIP (**a**), CH-HAS-LIP (**b**) and SA/CH-HAS-LIP (**c**).

Figure 2 presents the morphology of HAS-LIP, CH-HAS-LIP and SA/CH-HAS-LIP, which reveals the structure of a liposome. The obtained HAS-LIP (Figure 2a) was exposed as bright white, with homogeneously dispersed and spherical nano-particles. After being coated with CH and SA, the particle size of these formulations became larger. As shown in Figure 2b, CH-HAS-LIP obviously covered transparent multiple-layer materials, which might be CH forming the outer layer of HAS-LIP. The spherical and core-shell structure of SA/CH-HAS-LIP (Figure 2c) was also demonstrated. There was a ring of gray-white materials on the outer layer, and it was speculated that the gray-white materials were SA and CH [29]. This may be attributed to the polyelectrolytes (including CH and SA), which tend to bind with stained molecules outside nano-liposomes [30].

### 3.4. Differential Scanning Calorimetry

**Figure 3:**
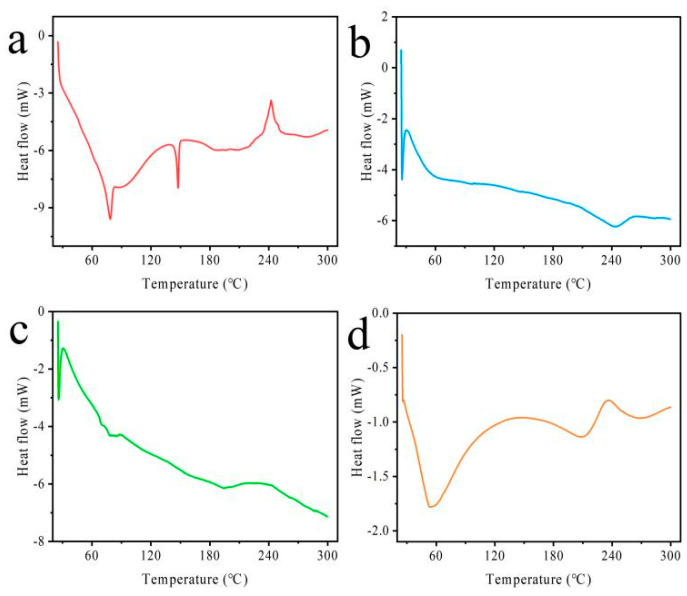
DSC curve of a physical mixture of excipients (**a**), HAS-LIP (**b**), CH-HAS-LIP (**c**) and SA/CH-HAS-LIP (**d**).

In Figure 3, the DSC thermograms of all excipients and formulations were obtained. Endothermic peaks were exhibited at 84 °C and 146 °C by HSPC and cholesterol, respectively, while HAS-LIP showed a prominent endothermic peak at 240 °C. It was reported that HAS showed an endothermic peak at 206 °C followed by dissociation [14]. There was no same HAS peak in the thermogram of HAS-LIP, indicating that HAS might exist in an amorphous state in the HAS-LIP dispersion. The polymers CH and SA exhibited peaks at 215 °C and 242 °C, respectively [31,32]. As shown in CH-HAS-LIP (Figure 3c), a broad endothermic peak at 235 °C might be associated with the hydrophobic or electrostatic interactions between liposomes and CH. Additionally, it was observed that the SA peak shifted towards a lower temperature (from 242 °C to 235 °C) in SA/CH-HAS-LIP. The decrease in temperature of the SA peak may be related to the electrostatic interactions between charged materials (such as SA and CH).

### 3.5. Fourier Transform Infrared Spectrum Analysis

**Figure 4:**
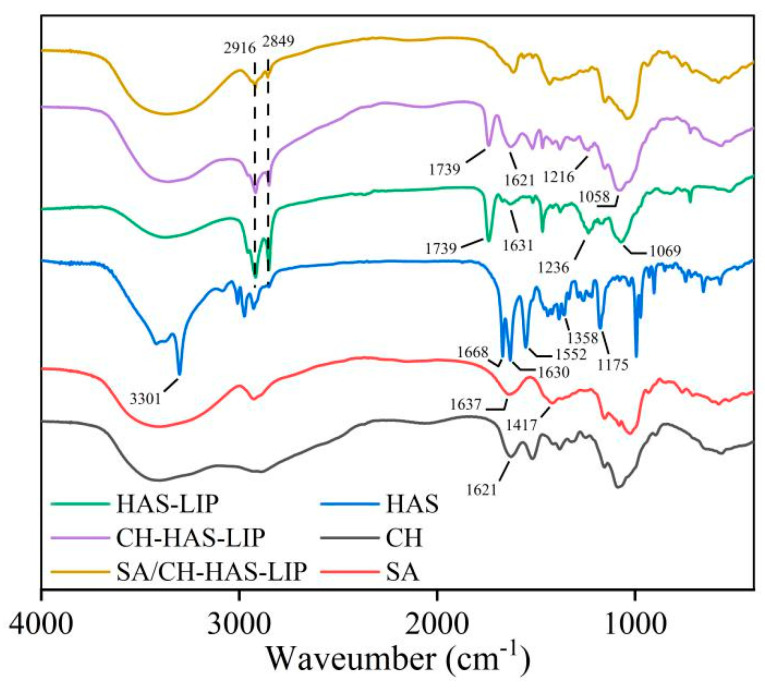
FTIR spectra of HAS, CH, SA, HAS-LIP, CH-HAS-LIP and SA/CH-HAS-LIP.

To investigate the possible interaction between polymers and liposomes, the FTIR spectra of HAS, CH, SA, HAS-LIP, CH-HAS-LIP and SA/CH-HAS-LIP are shown in Figure 4. HAS had a characteristic peak at 3301 cm^−1^, which overlapped the O-H and N-H stretching vibration. The peaks at 1668, 1552 and 1175 cm^−1^ were associated with the C=O stretching vibration in amide I, the N-H bending vibration in amide II and the C-O stretching vibration, respectively [33]. In the spectra of HAS-LIP, an absorption peak at 1631 cm^−1^ was observed, which was the stretching band for C=C groups of HAS. The spectra of all liposomes exhibited the same -CH_2_- group signals at 2916 and 2849 cm^−1^ but with some differences in these peaks’ intensity. This might demonstrate that the structure of HAS-LIP was not destroyed after surface modification.

In the FTIR spectrum of CH-HAS-LIP, the intensity of the peak at 1739 cm^−1^ (C=O stretching) corresponding to liposomal phospholipids in HAS-LIP obviously decreased. Meanwhile, the peaks of PO^2−^ groups in HAS-LIP at 1236 and 1069 cm^−1^ shifted to 1216 and 1058 cm^−1^, respectively, which further displayed a strong hydrogen-bonding interaction between CH and HAS-LIP [34].

The absorption peak at 1621 cm^−1^ was assigned to the N-H bending of -NH^3+^ groups of CH, whereas the characteristic peaks of SA at 1637 and 1417 cm^−1^ corresponded to the asymmetric and symmetric stretching of -COO^−^ groups, respectively. However, there were no absorption peaks at 1637, 1417 and 1621 cm^−1^ in the SA/CH-HAS-LIP spectra. This suggests that -NH^3+^ groups on CH could react with -COO^−^ groups on SA by electrostatic attraction to deposit a polyelectrolyte shell on the surface of HAS-LIP [35]. Additionally, the interactions between amide groups of CH and carboxylate groups of SA could form hydrogen bonding between the polymers [36]. The results, demonstrated that HAS-LIP was successfully covered with CH and SA layer by layer.

### 3.6. In Vitro Release Study

**Figure 5:**
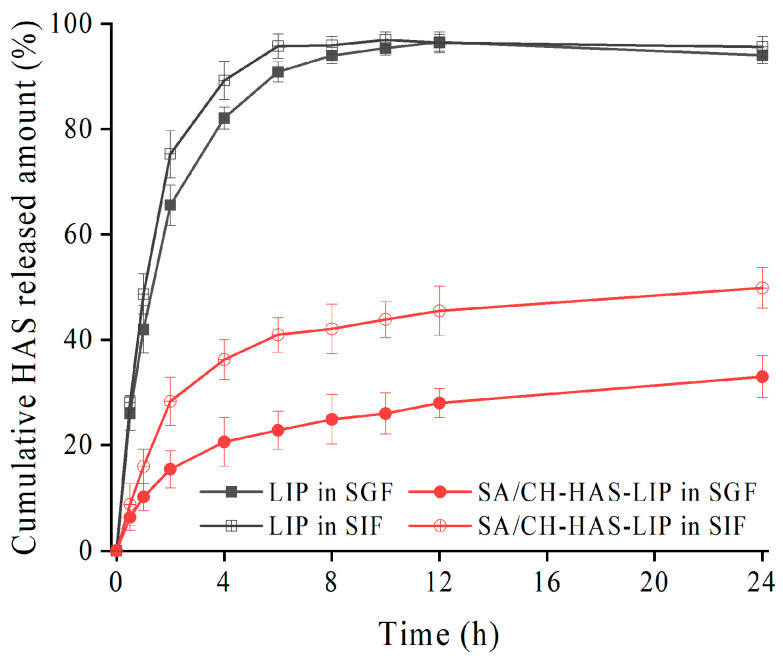
Drug release curve of HAS-LIP and SA/CH-HAS-LIP in SGF or SIF (*n* = 3).

To characterize the release performance of different liposome formulations, the release of HAS from HAS-LIP and SA/CH-HAS-LIP was performed in SGF or SIF. As shown in Figure 5, drug release from HAS-LIP was more rapid than that of SA/CH-HAS-LIP in SGF. It was observed that drug release from HAS-LIP (42.45 ± 4.52%) in SGF was higher than that from SA/CH-HAS-LIP (22.84 ± 3.68%) within 1 h. A total of 90.87 ± 1.95% of the drug from HAS-LIP in SGF was released within 4 h, while 20.62 ± 4.67% of HAS was released from SA/CH-HAS-LIP. Similarly, HAS-LIP presented about 48% of drug release in SIF in 1 h and over 95% after 24 h, whereas SA/CH-HAS-LIP showed about 15% of release in SIF in 1 h and about 49% after 24 h.

It was clear that faster and higher release rates were observed in HAS-LIP compared with coated liposomes in both SGF and SIF. The HAS-LIP formulation failed to delay drug release under acidic conditions that commonly exist in the stomach due to the hydrolysis of phospholipids [37]. On the contrary, the release behavior of SA/CH-HAS-LIP displayed obvious pH dependence. When exposed to the acid environment, there was less initial burst release in SA/CH-HAS-LIP. The core-shell structure of SA/CH-HAS-LIP could decrease the permeability of the lipid bilayer and protect the liposomal membrane from degradation. This may be attributed to the hard shell induced by the intermolecular hydrogen bond force between polymers under low pH conditions and the ability of SA to reduce the activity of digestive enzymes [38]. The delayed release of the drug from SA/CH-HAS-LIP might demonstrate that HAS was more likely to be delivered to the intestine. This suggests that the modified liposome has potential application in oral administration to promote the oral bioavailability of lipophilic drugs in vivo. In a neutral environment, the drug release of SA/CH-HAS-LIP showed initial burst release due to the dissolution of outer-layer polymers (SA) at a pH of 6.8. After that, the coated liposome showed distinct controlled-release characteristics up until 24 h in comparison to HAS-LIP, which might indicate that SA/CH-HAS-LIP has great potential to provide sustained drug delivery during treatment. It was speculated that the structural integrity of liposomes might still be hardly destroyed due to the insolubility of CH (the inner layer) at a pH of 6.8 [39].

To further investigate the HAS release mechanism, the best fitted release model was selected according to the correlation coefficient (R^2^). The equations given below were used to establish the drug mechanism of drug transport from different liposome formulations:

(3) $$M_{t}=M_{\infty }\times \left\{1-\exp(-kt)\right\},$$

(4) $$M_{t}=M_{\infty }\times \left\{1-\exp(-at^{b})\right\},$$

where *M_t_* is the drug release at time *t*, *M_∞_* is the total amount of drug loaded, *t* is the release time, *k* is the first-order rate constant and *a* and *b* are Weibull release parameters.

As shown in Table 2, the data of HAS-LIP in two digestive juices were fitted well to the first-order model (R^2^ > 0.99). It suggested that the drug release was governed by diffusion through a lipid matrix due to the micropore structure of liposomes. For SA/CH-HAS-LIP in SGF or SIF, the Weibull model was used to describe the release mechanism (R^2^ was 0.9986 and 0.998, respectively). The Weibull model is an empiric model and is often discussed in combination with other release models [40]. It could be seen that the R^2^ values of the first-order model (R^2^ = 0.9887), Higuchi model (R^2^ = 0.9759) and Weibull model for SA/CH-HAS-LIP in SGF were close. Based on the Weibull equation in Table 3, with b values ranging between 0.39 and 0.69, SA/CH-HAS-LIP in SGF was found to follow a Fickian diffusion mechanism. The drug release of SA/CH-HAS-LIP was driven by diffusion through the fractal or disordered matrix slowly due to the core-shell structure [41]. With b values ranging from 0.69 to 0.75, SA/CH-HAS-LIP in SIF indicated a complex release mechanism, probably including erosion-based release and drug diffusion [42].

### 3.7. In Vivo Pharmacokinetics

**Figure 6:**
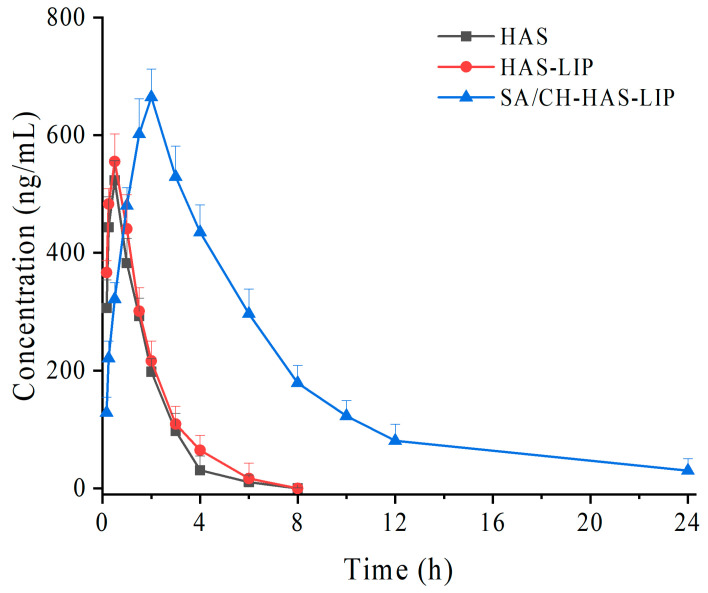
Concentration time curves of different samples after administration in rat plasma (*n* = 6).

To further assess the influence of the modified liposomes on the transport and absorption of the drug in vivo, the plasma concentration-time curves of HAS after the intragastric administration of free HAS and SA/CH-HAS-LIP are shown in Figure 6. The pharmacokinetic analysis was performed using a two-compartmental method and is summarized in Table 4. As result, the plasma concentration of HAS of free drug groups immediately increased after 0.167 h (>300 ng/mL) and decreased after 4 h (<30 ng/mL). The t_1/2α_ and t_1/2β_ of HAS were at 0.67 ± 0.33 h and 1.53 ± 0.36 h after intragastric administration, respectively, indicating that HAS was rapidly absorbed and eliminated within 4 h. There was no significant difference in t_1/2α_, t_1/2β_, t_max_, AUC_(0-t)_ and AUC_(0-∞)_ between HAS and HAS-LIP groups, suggesting that drug-loaded liposomes did not affect the pharmacokinetic behavior of HAS. This might be due to the disruption of the structural integrity of liposomes in the stomach quickly, which was in agreement with the in vitro release study.

However, for SA/CH-HAS-LIP groups, all pharmacokinetic parameters were significantly raised (*p* > 0.05, *p* > 0.01) in comparison with HAS or HAS-LIP groups. The t_1/2α_, t_1/2β_ and t_max_ of SA/CH-HAS-LIP groups remarkably extended to 1.95 ± 0.67 h, 9.29 ± 0.75 h and 2.00 ± 0.47 h, respectively, which revealed a sustained-release characteristic of SA/CH-HAS-LIP. In addition, the C_max_ values of coated liposomes were higher than those of free drug and uncoated liposome groups. This is probably because the pH-sensitive outer layer of SA/CH-HAS-LIP could efficiently reduce the initial burst release and undergo a relatively lesser degradation or release of HAS during the passage through the stomach.

When compared with the HAS and HAS-LIP groups, the AUC of SA/CH-HAS-LIP also significantly raised by 4.6-fold and 4.2-fold, respectively. This illustrates that the oral bioavailability of HAS was obviously improved. The increase in oral bioavailability of the drug in SA/CH-HAS-LIP might be related to multiple factors, such as the following: (a) the core-shell drug carrier played an important role in the absorption rate of the drug due to its high stability and sustained-release characteristics in the gastrointestinal tract; (b) the biocompatible materials (such as HSPC, CH and SA) might improve the permeability of the gastrointestinal membrane and the cellular absorption of the drug-loaded liposome [43]; (c) the mucoadhesive property of CH could effectively increase the interaction between the coated liposome and the gastrointestinal membrane, resulting in the extended time of HAS at the absorption site [44]; (d) with the dissolution of SA, part of the coated liposome (smaller than 500 nm) may be diffused through the mucosal barrier or M-cells in the gastrointestinal tract [45]. Moreover, the MRT values for SA/CH-HAS-LIP groups were four times higher than those for the free drug or HAS-LIP groups, which might reflect that the coated liposome could extend the residence time of HAS in the systemic circulation.

## 4. Conclusions

In summary, this work investigated the potential of a novel delivery system to improve the oral bioavailability of HAS. After coating HAS-LIP with CH and SA layer-by-layer, the obtained SA/CH-HAS-LIP reflected good drug loading capacity and exhibited a sustained release behavior. In addition, the pH-dependence of SA/CH-HSA-LIP was observed, which demonstrated that HAS was more absorbed in the intestine. Compared with free HAS and HAS-LIP, SA/CH-HAS-LIP could significantly improve the oral bioavailability of HAS (4.6-fold and 4.2-fold) in vivo. Therefore, this novel delivery system could be considered as a promising release carrier for the oral delivery of the drug to enhance the solubility and bioavailability of HAS.

## Figures and Tables

**Table 1:** Particle size, PDI, zeta potential entrapment efficiency and drug loading of HAS-LIP, CH-HAS-LIP and SA/CH-HAS-LIP (*n* = 3).

Formulation	Particle Size (nm)	PDI	Zeta Potential (mV)	Encapsulation Efficiency (%)	Drug Loading (%)
HAS-LIP	107.81 ± 2.11	0.15 ± 0.05	−58.20 ± 3.4	96.13 ± 1.77	3.41 ± 0.47
CH-HAS-LIP	212.32 ± 7.73	0.26 ± 0.07	56.10 ± 4.29	93.52 ± 3.95	3.12 ± 0.31
SA/CH-HAS-LIP	533.71 ± 12.39	0.37 ± 0.16	−49.40 ± 6.72	92.39 ± 4.74	2.94 ± 0.62

**Table 2:** The correlation coefficient of five release kinetic models of HAS-LIP and SA-CH-HAS-LIP in SGF and SIF.

Model	SGF		SIF	
	HAS-LIP	SA/CH-HAS-LIP	HAS-LIP	SA/CH-HAS-LIP
Zero equation	0.6899	0.7780	0.7139	0.7262
First-order equation	0.9975	0.9887	0.9999	0.9973
Higuchi equation	0.9179	0.9759	0.9214	0.9519
Ritger-peppas equation	0.4876	0.9674	0.8504	0.8707
Weibull equation	0.9891	0.9986	0.9899	0.9981

**Table 3:** Release kinetic equations of HAS-LIP and SA/CH-HAS-LIP in SGF or SIF.

Formulation	SGF	SIF
HAS-LIP	$M_{t}=95.02\left(1-e^{-0.55t}\right)$	$M_{t}=96.47\left(1-e^{-0.70t}\right)$
SA/CH-HAS-LIP	$M_{t}=35.22\left(1-e^{-0.34t^{0.63}}\right)$	$M_{t}=48.70\left(1-e^{-0.43t^{0.83}}\right)$

**Table 4:** The concentration of plasma of different samples after administration in rat plasma (*n* = 6).

Parameter	Unit	HAS	HAS-LIP	SA/CH-HAS-LIP
t_1/2α_	h	0.67 ± 0.33	0.51 ± 0.35	1.95 ± 0.67 *
t_1/2β_	h	1.53 ± 0.36	1.85 ± 1.51	9.29 ± 0.75 **
t_max_	h	0.50 ± 0.17	0.50 ± 0.13	2.00 ± 0.47 **
C_max_	ng/mL	523.25 ± 26.94	555.26 ± 38.38	664.49 ± 39.19 **
AUC_(0-t)_	ng/mL·h	948.77 ± 115.60	1030.63 ± 133.04	4367.02 ± 425.49 **
AUC_(0-∞)_	ng/mL·h	1046.68 ± 122.46	1084.37 ± 134.27	4532.57 ± 446.41 **
MRT_(0-t)_	h	1.36 ± 0.10	1.44 ± 0.13	6.10 ± 0.19 **
MRT_(0-∞)_	h	1.53 ± 0.11	1.69 ± 0.15	7.27 ± 0.31 **

* Means significant, ** means very significant.

## Data Availability

Almost all data are presented within the manuscript (figures and tables). The raw data presented in this study are available upon request to the corresponding author.

## Supplementary Materials

The following supporting information can be downloaded at: https://www.mdpi.com/article/10.3390/pharmaceutics15072010/s1, Figure S1: Particle size (a), and zeta potential (b) of HAS-LIP, CH-HAS-LIP and SA/CH-HAS-LIP; Table S1: Optimization of concentrations of CH and SA for preparation of SA/CH-HAS-LIP.
