# Supplementary material for: Sodium Alginate/Chitosan-Coated Liposomes for Oral Delivery of Hydroxy-α-Sanshool: In Vitro and In Vivo Evaluation
Source: Pharmaceutics. 2023 Jul 24;15(7):2010. doi: 10.3390/pharmaceutics15072010 (PMC10383520; doi:10.3390/pharmaceutics15072010)
Supplement: Supplementary file 1 [file pharmaceutics-15-02010-s001.zip › pharmaceutics-2482328-supplementary.pdf]

Supplementary Method

Optimization of SA/CH-HAS-LIP formulation

Supplementary Table S1 Optimization of concentrations of CH and SA for preparation of SA/CH-HAS-LIP

Supplementary Figure S1 Particle size (a), and zeta potential (b) of HAS-LIP, CH-HAS-LIP and SA/CH-HAS-LIP

Supplementary Method

Optimization of SA/CH-HAS-LIP formulation

The concentrations of CH and SA were optimized by the parameters of particle size, PDI, zeta potential, encapsulation efficiency, and sedimentation efficiency, respectively.

As the results shown in Table S1, a similar EE% was obtained from CH-HAS-LIP formulation in the cases of 0.6% and 0.8% CH. However, some flocculation could be found in the liposome coated with 0.8% of CH after storage for 48 h. Therefore, 0.6% of CH was selected as the first coating layer of HAS-LIP in the following experiments. Besides, compared to the other concentrations of SA, the liposome coated with 0.4% of SA exhibited small particle size, positive zeta potential value and low sedimentation efficiency. Hence, the appropriate concentration of SA for the following coating purpose was 0.4%.

Table S1. Optimization of concentrations of CH and SA for preparation of SA/CH-HAS-LIP

	Concentrations of polymer (%, w/v)	Particle size (nm)	Zeta potential (mV)	Encapsulation efficiency (%)	Sedimentation efficiency (%)
CH-HAS-LIP	0.2	135.13 ± 5.74	38.6 ± 0.6	84.13 ± 1.85	
	0.4	175.95 ± 6.95	40.2 ± 0.6	89.40 ± 1.30	
	**0.6**	**214.89 ± 4.93**	**53.7 ± 0.4**	**96.17 ± 1.84**	
	0.8	304.84 ± 8.69	60.9 ± 0.3	95.88 ± 1.53	
	1.0	543.01 ± 10.48	65.5 ± 0.4	95.18 ± 1.23	
	2.0	680.88 ± 15.91	68.8 ± 0.2	96.01 ± 1.06	
SA/CH-HAS-LIP	0.1	353.73 ± 11.68	41.0 ± 0.2		2.92 ± 0.09
	0.2	447.03 ± 8.96	29.7 ± 0.3		7.30 ± 0.07
	**0.4**	**533.71 ± 12.39**	**-41.7 ± 0.2**		**28.49 ± 0.15**
	0.6	815.95 ± 15.61	-65.5 ± 0.2		40.09 ± 0.24
	0.8	2763 ± 23.23	-70.9 ± 0.1		57.9 ± 0.23
	1.0	3615 ± 10.75	-74.2 ± 0.1		62.31 ± 0.16

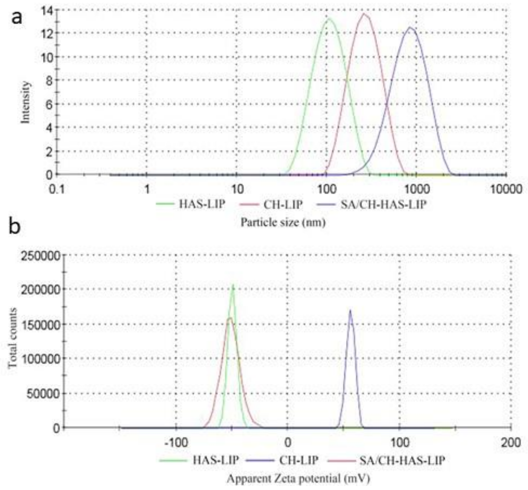

Figure S1. Particle size (a), and zeta potential (b) of HAS-LIP, CH-HAS-LIP and SA/CH-HAS-LIP
